# Development and external validation of a dynamic nomogram to predict the survival for adenosquamous carcinoma of the pancreas

**DOI:** 10.3389/fonc.2022.927107

**Published:** 2022-08-12

**Authors:** Chao Ren, Yifei Ma, Jiabin Jin, Jiachun Ding, Yina Jiang, Yinying Wu, Wei Li, Xue Yang, Liang Han, Qingyong Ma, Zheng Wu, Yusheng Shi, Zheng Wang

**Affiliations:** ^1^ Department of Hepatobiliary and Pancreatic Surgery, The Affiliated Jinhua Hospital of Zhejiang University School of Medicine, Jinhua, China; ^2^ Department of Hepatobiliary Surgery, The First Affiliated Hospital of Xi’an Jiaotong University, Xi’an, China; ^3^ Department of General Surgery, Pancreatic Disease Center, Ruijin Hospital, Shanghai Jiaotong University School of Medicine, Shanghai, China; ^4^ Department of Pathology, The First Affiliated Hospital of Xi’an Jiaotong University, Xi’an, China; ^5^ Department of Medical Oncology, The First Affiliated Hospital of Xi’an Jiaotong University, Xi’an, China

**Keywords:** adenosquamous carcinoma, pancreas, nomogram, prognosis, the TNM 8^th^ staging system

## Abstract

**Objective:**

We aimed to develop a nomogram to predict the survival and prognosis of adenosquamous carcinoma of the pancreas (ASCP).

**Background:**

Adenosquamous carcinoma of the pancreas (ASCP) is a relatively rare histological subtype of pancreatic exocrine neoplasms. It was reported a worse survival in ASCP than in pancreatic adenocarcinoma (PDAC). Prediction of ASCP prognosis is of great importance.

**Methods:**

Histologically confirmed ASCP patients from the National Cancer Institute’s Surveillance, Epidemiology, and End Results (SEER) Program database were finally enrolled and divided into development and internal validation cohorts. Moreover, a multi-center cohort of 70 patients from China was registered as the external validation. A nomogram was developed based on independent predictors of ASCP determined in multivariable analysis.

**Results:**

A total of 233 patients from SEER were finally included. Univariate and Multivariate analysis showed that tumor size, radiotherapy, chemotherapy, and lymph node ratio (LNR) were considered the independent prognostic indicators. We developed a nomogram according to these four parameters. The C index of the nomogram in the development cohort was 0.696. Through analysis of the area under the curve (AUC) of the different cohorts, we observed that the predictive efficacy of the nomogram for 1-, and 2-year overall survival (OS) were better than those of the American Joint Committee on Cancer (AJCC) TNM (8^th^) staging system both in the development and validation cohort. External validation confirmed that 1-year survival is 67.2% vs. 29.7%, similar to the internal cohort analysis.

**Conclusion:**

The nomogram showed good performance in predicting the survival of ASCP. It could help surgeons to make clinical decisions and develop further plans.

## Highlights

- We developed a nomogram especially for adenosquamous carcinoma of the pancreas (ASCP) to predict the survival and prognosis.
- Univariate and multivariate analysis showed that four parameters, including tumor size, radiotherapy, chemotherapy, and lymph node ratio (LNR), could influence the survival of ASCP patients.
- In addition, by comparing the nomogram’s efficiency with AJCC TNM (8^th^) staging system, it was proved that the nomogram is superior to the TNM stage in predicting the prognosis.
- Finally, we applied the external cohort from multi-center to verify the performance and get satisfactory results. Our nomogram has clinical applicability.

## Introduction

Adenosquamous carcinoma of the pancreas (ASCP) is a relatively rare histological subtype of pancreatic exocrine neoplasms, comprising at least 30% malignant squamous cell carcinoma mixed with ductal adenocarcinoma (1). Unlike pancreatic ductal adenocarcinoma (PDAC), it accounts for 1-4% of exocrine pancreatic malignancies and is considered an enigmatic and aggressive tumor with a worse prognosis and higher metastatic potential than its adenocarcinoma counterpart (2, 3). Previously a large population-based cohort of 415 patients with ASCP focused on a significant difference between the survival of patients with ASCP and those with pancreatic adenocarcinoma (PDAC) after resection, with median OS of 12 and 16 months, respectively, which showed a worse survival in ASCP than patients with PDAC (4). The majority of the available series report a poor prognosis for patients with ASCP, which might result in a therapeutic nihilism with the omission of potentially curative multimodal therapy (5, 6).

Though the 8^th^ edition of the American Joint Committee on Cancer (AJCC) and TNM stage are diagnosed for pancreatic cancer, it mainly accounts for PDAC and may not represent the actual situation of ASCP (7). Given the different histology characteristics between ASCP and PDAC, it is necessary to find another predictor technique for ASCP. Here we aimed to develop nomograms to evaluate the outcome of ASCP patients. We hypothesized that a combination of baseline characteristics and surgical information could improve the evidence-based selection of candidates and aid clinical decisions. In addition, we applied a multi-center cohort to confirm the effect of nomograms.

## Methods

### Patient and date collection

The SEER database (2004–2016) was used to identify the ASCP patients. Patients were retrieved based on the International Classification of Disease for Oncology (ICD, 3rd edition) codes for pancreas tumours. In order to identify all eligible cases, the following criteria were applied: (i) all patients were diagnosed as ASCP (ICD-O-3: 8560/3) (4, 7) with surgical resection and pathology verified. (ii) active follow-up of patients (diagnosis not obtained from autopsy or death certificate) (iii) positive histology confirmation and surgical resection (iv) Complete data of tumor size, lymph nodes examined, and positive lymph nodes.

Two Chinese centers provided external validation data on adenosquamous carcinoma of the pancreas. Data from the following centers included Ruijin Hospital, Shanghai Jiaotong University (n = 52), and The First Affiliated Hospital of Xi’an Jiaotong University (n = 18). Electronic datasheets were provided to the two centers. The Institutional Review Boards of the First Affiliated Hospital of Xi’an Jiaotong University approved all the ethical information (XJTU1AF2021LSK-053).

### Variables included

In the study, the following characteristics were reviewed: age, race, sex, tumor location, grade, AJCC TNM (8^th^) staging, T classification, N classification, M classification, radiotherapy, chemotherapy, number of lymph nodes examined, lymph nodes positive, tumor size, lymph node ratio (LNR), survival months, and vital status. The LNR is defined as the ratio of the number of metastatic lymph nodes relative to the total number of LNs examined (TNLE). The receiver-operating characteristic curve (ROC) analysis was used to investigate the discriminatory power relative to overall survival among patients who had 1 to 3 lymph node metastasis (LNM) and patients who had ≧4 LNM.

### Statistical analysis

All statistical analyses were performed by SPSS 25.0 statistical package (IBM Corporation, Armonk, NY, USA). The optimal cutoff value of the lymph nodes ratio and other parameters were analyzed by X-tile software. Continuous data were expressed in medians with interquartile range (IQR), and Mann–Whitney U tests were used to comparing these data. Categorical data were compared using χ2 or Fisher exact tests. The overall survival (OS) was compared by Kaplan-Meier curves and analyzed using the log-rank test *via* SPSS and GraphPad Prism 8.0 Software (GraphPad Software Inc. San Diego, CA, USA). The Cox proportional hazards regression models were performed to find the independent prognostic factors. The cutoff values of the variables were determined by X-tile software (Yale University, New Haven, CT, USA) (8). The resulting hazard ratios (HR) and 95% confidence interval (CI) were presented. All tests were two-sided, and *P*-value < 0.05 was considered statistically significant.

### Nomogram establishment

We divided the whole cohort from SEER into development and internal validation cohorts at a ratio of 7:3 using a table of random numbers. Based on the results of multivariate Cox regression in the development cohort, potential risk factors (*P* < 0.05) were used to establish a nomogram using the “rms” R package. The accuracy and calibration of the model were verified using the bootstrap verification method and consistency index (C-index). The closer the C-index to 1, the better the model discrimination. The closer the calibration curve of the graph calibration method is to the standard curve (slope 1), the better the predictive ability of the nomogram is. The R language software version used for the study was version 3.5.1. Additionally, we applied a multi-center cohort from China as the external cohort to confirm nomogram efficiency. The authors have completed the STROBE Statement of cohort studies checklist (9).

## Results

### Clinicopathological characteristics

According to the criteria above, 233 patients with histologically confirmed pancreatic adenosquamous carcinoma from the SEER database were finally included (**Supplement Figure 1**). There were 115 males and 118 females, with a median age of 68 (60–74). Tumors were located at the pancreatic head (116/233, 49.79%) and the body or tail (91/233, 39.06%). 56 patients (56/233, 24.03%) received radiotherapy, while 149 (149/233, 63.95%) received chemotherapy. The median number of examined lymph nodes was 15 (9–22), and the median number of tumor size was 40 (30–55) mm. A development set of SEER database (n=165) and validation set (n=68) were analyzed. The detailed baseline characteristics are displayed in **Tables 1**, **2**.

**Table 1:** The clinical characteristics of the ASCP patients from SEER and a multi-center cohort.

Characteristics	SEER database (N = 233)	Chinese centers (N = 70)	*P* value
Diagnosed age	68 (60–74)	61 (53-70)	< 0.001
Race		NA^a^	
Black	22	/	
White	193	/	
Others	18	/	
Sex			0.016
Female	118	24	
Male	115	46	
Tumor location			0.010
Body or tail	91	27	
Head	116	43	
Others	26	/	
Grades		NA^a^	/
I+II	54	/	
III+IV	149	/	
Unknown	30	/	
AJCC stages (8^th^)			0.212
I	55	23	
II	125	36	
III	53	11	
T Stage (8^th^)			0.203
T1	11	7	
T2	109	33	
T3	98	23	
T4	15	7	
N Stage (8^th^)			0.049
N0	95	39	
N1	97	25	
N2	41	6	
Radiotherapy			< 0.001
No	177	70	
Yes	56	0	
Chemotherapy			0.021
No	84	36	
Yes	149	34	
Number of examined lymph nodes	15 (9-22)	12 (6-18.3)	0.012
Tumor size	40 (30-55)	40 (30-60)	0.673
LNR	0.071 (0-0.20)	0.00 (0-0.10)	0.011

a No relevant data have been collected.

ASCP, adenosquamous carcinoma of the pancreas; SEER, the National Cancer Institute’s Surveillance, Epidemiology, and End Results; AJCC, American Joint Committee on Cancer; LNR, Lymph node ratio.

**Table 2:** Clinical characteristics of the ASCP patients from SEER in the development and internal validation cohorts.

Characteristics	Development cohorts (N = 165)	Internal validation cohorts (N = 68)
Diagnosed age	67 (59-73)	70 (63-75)
Race		
Black	16	6
White	139	54
Others	10	8
Sex		
Female	91	27
Male	74	41
Tumor location		
Body or tail	67	24
Head	79	37
Others	19	7
Grades		
I+II	39	15
III+IV	102	47
Unknown	24	6
AJCC stages (8^th^)		
I	37	18
II	95	30
III	33	20
T Stage (8^th^)		
T1	8	3
T2	76	33
T3	71	27
T4	10	5
N Stage (8^th^)		
N0	69	26
N1	72	25
N2	24	17
Radiotherapy		
No	119	58
Yes	46	10
Chemotherapy		
No	59	25
Yes	106	43
Number of examined lymph nodes	14 (9-22)	16 (11-22.75)
Tumor size	40 (30-55)	40 (30-50)
LNR	0.070 (0-0.185)	0.007(0-0.208)

The optimal cutoff value of the lymph nodes ratio analyzed by X-tile software was 0.18 (**Supplement Figure 2**). Receiver-operating characteristic (ROC) analysis illustrates that the total number of lymph nodes examined (TNLE) ≧16 had the highest discriminatory power relative to overall survival among patients who had 1 to 3 lymph node metastasis (LNM) and patients who had ≧4 LNM (10, 11) (AUC 0.775, Youden index 0.434, sensitivity 80.5%, specificity 62.9%, *P* < 0.001)(**Supplement Figure 3**). Above all, we choose 16 as the cutoff value of the number of lymph nodes examined.

In addition, 70 patients from the two centers in China were also included, as **Table 1** shows. There were 46 males and 24 females were diagnosed with ASPC from 2012 to 2019. The median age at diagnosis was 61 (53–70). The tumor was located at the pancreatic head (n = 43) and pancreatic body or tail (n = 27). The median number of examined lymph nodes was 12 (6–18), and the median number of tumor size was 40 (30–60) mm. All the patients did not receive radiotherapy, while 34 patients received chemotherapy.

### Univariate and multivariate analysis on independent prognostic factors for the prognosis of ASCP from SEER

To further analyze clinical characteristics of the survival and prognosis of patients with pancreatic adenosquamous carcinoma, we firstly conducted univariate and multivariate analyses of the overall survival (OS) of patients with ASCP from the SEER database. A total of 233 patients were analyzed with the single-factor Cox regression. According to the results, age, tumor size, AJCC stage, N stage, chemotherapy, radiotherapy, and LNR were all related to the prognosis of patients with ASCP (*P* < 0.05) (**Table 3**).

**Table 3:** Univariate analysis of clinical characteristics in ASCP patients.

Characteristics	N (%)	Univariate analysis		
		HR	95% CI	*P* value
Diagnosed age				
≤ 68	121 (51.9%)	1	/	/
> 68	112 (48.1%)	1.366	1.011-1.845	0.042
Race				
Black	22 (9.4%)	1	/	/
White	193 (82.8%)	0.661	0.404-1.081	0.099
Others	18 (7.8%)	0.654	0.319-1.344	0.248
Sex				
Female	118 (50.6%)	1	/	/
Male	115 (49.4%)	1.072	0.793-1.448	0.652
Tumor location				
Body or tail	91 (39.1%)	1	/	/
Head	116 (49.8%)	1.182	0.859-1.627	0.305
Others	26 (11.1%)	1.234	0.747-2.039	0.412
Grades				
I+II	54 (23.2%)	1	/	/
III+IV	149 (63.9%)	1.084	0.753-1.560	0.665
Unknown	30 (12.9%)	1.035	0.608-1.761	0.899
AJCC stages (8^th^)				
I	55 (23.6%)	1	/	/
II	125 (53.6%)	1.080	0.737-1.582	0.694
III	53 (22.8%)	1.787	1.148-2.783	0.010
T Stage (8^th^)				
T1	11 (4.7%)	1	/	/
T2	109 (46.8%)	0.790	0.380-1.639	0.526
T3	98 (42.1%)	1.082	0.521-2.244	0.833
T4	15 (6.4%)	1.521	0.630-3.675	0.351
N Stage (8^th^)				
N0	95 (40.8%)	1	/	/
N1	97 (41.6%)	1.296	0.928-1.810	0.128
N2	41 (17.6%)	1.838	1.208-2.796	0.004
Radiotherapy				
No	177 (76.0%)	1	/	/
Yes	56 (24.0%)	0.475	0.330-0.686	<0.001
Chemotherapy				
No	84 (36.1%)	1	/	/
Yes	149 (63.9%)	0.444	0.329-0.600	< 0.001
Number of examined lymph nodes				
1-15	125 (53.6%)	1	/	/
≥ 16	108 (56.4%)	1.013	0.749-1.370	0.934
Tumor size				
< 40mm	101 (43.3%)	1	/	/
≥ 40mm	132 (56.7%)	1.472	1.084-1.998	0.013
LNR				
< 0.18	170 (73.0%)	1	/	/
≥ 0.18	63 (27.0%)	1.663	1.202-2.301	0.002

ASCP, adenosquamous carcinoma of the pancreas; HR, hazard ratio; CI, confidence interval; AJCC, American Joint Committee on Cancer; LNR, Lymph node ratio.

According to the results of single factor analysis and professional conclusions, Cox proportional risk regression analysis was further conducted. As showed in **Table 4**, multivariate analysis indicated that tumor size (*P* = 0.004, HR = 1.573, 95% CI: 1.156 to 2.240), radiotherapy (*P* = 0.016, HR = 0.617, 95% CI: 0.416 to 0.914), chemotherapy (*P* < 0.001, HR = 0.511, 95% CI: 0.373 to 0.700), and LNR (*P* = 0.019, HR = 1.488, 95% CI: 1.068 to 2.074) were considered independent prognostic indicators for OS of patients with ASCP after surgical resection.

**Table 4:** Multivariate analysis of different influencing factors in ASCP patients.

Variables	B value	SE value	Wald value	*P* value	HR	95% CI
Radiotherapy	-0.483	0.201	5.803	0.016	0.617	0.416 - 0.914
Chemotherapy	-0.672	0.161	17.414	< 0.001	0.511	0.373 - 0.700
LNR	0.398	0.169	5.512	0.019	1.488	1.068 - 2.074
Tumor size	0.453	0.157	8.299	0.004	1.573	1.156 - 2.140

ASCP, adenosquamous carcinoma of the pancreas; HR, hazard ratio; CI, confidence interval; LNR, Lymph node ratio.

### Development and validation of a nomogram for predicting ASCP survival

**Figure 1:**
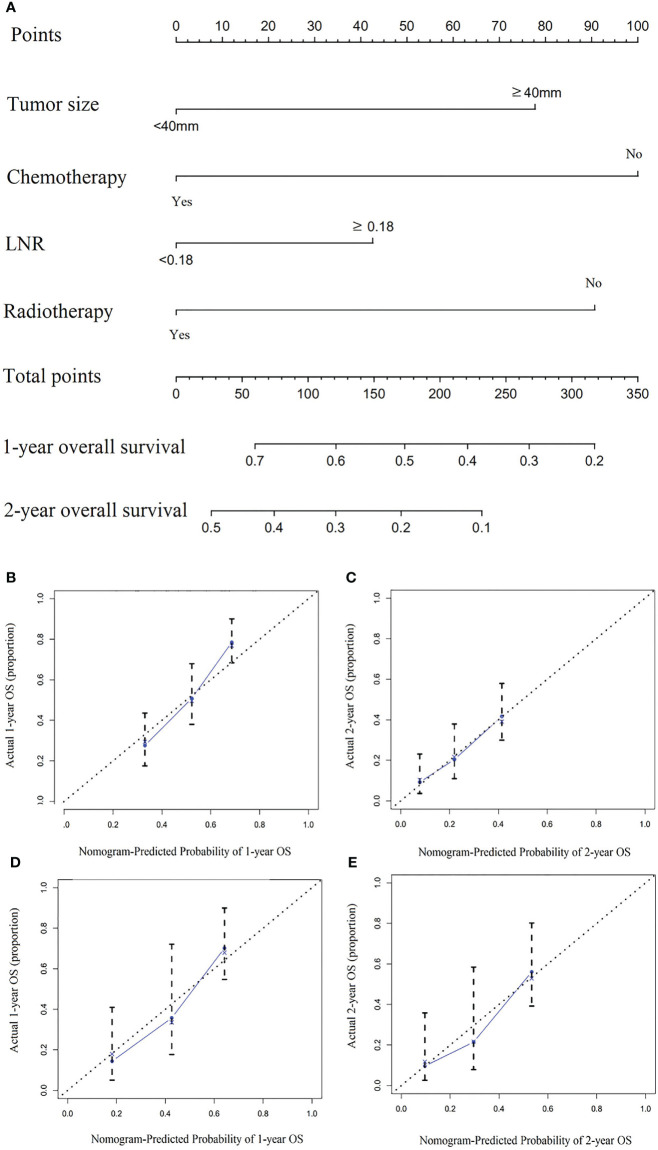
**(A)** The nomogram for predicting the overall survival of patients with pancreatic adenosquamous carcinoma. **(B–E)** The 1-year and 2-year calibration curves of the development group and the internal verification group for prognostic nomogram of patients with pancreatic adenosquamous carcinoma **(B)** 1-year development group **(C)** 2-year development group **(D)** 1-year internal verification group **(E)** 2-year internal verification group.

The nomogram included all statistically significant prognostic factors in the Cox proportional hazards regression model, including radiotherapy, chemotherapy, LNR, and tumor size (**Figure 1A**). Its influence on prognosis determined the score of each parameter, and the survival rate of the patients was obtained by the sum of the score of four parameters. To simplify applying the model in clinical practice, we also transformed the nomogram into a web-based calculator (https://aliez2021.shinyapps.io/DynNomapp/) (**Supplement Figure 4**).

To verify the efficiency of the established nomogram, we applied the bootstrap method. The C index of the nomogram in predicting survival of the development cohort was 0.696 (95% CI: 0.643-0.749). The C index in the internal validation cohort was 0.696 (95% CI: 0.617-0.776). The 1-year AUC of the development and internal validation cohort was 0.750 and 0.717, compared with 0.703 and 0.717 in 2 year AUC. The development group’s 1-year and 2-year calibration curves and the internal validation group did not deviate from the centerline, showing good prediction compliance (**Figures 1B–E**). These results showed good agreement between prediction and observation.

### Comparison of the nomogram and AJCC TNM (8^th^) staging system

**Figure 2:**
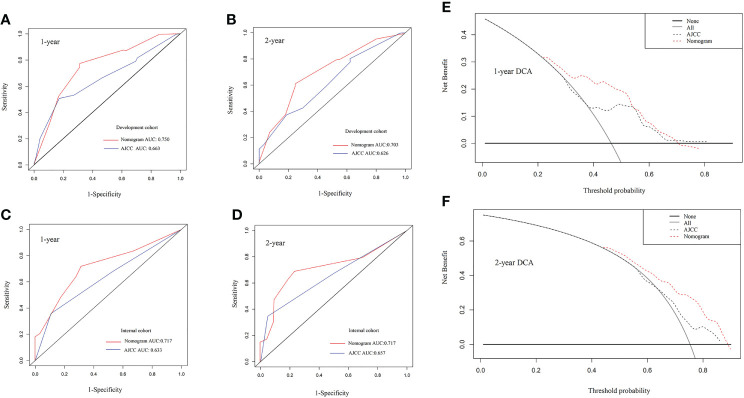
**(A–D)** The 1-year and 2-year receiver-operating characteristic (ROC) curve of the development group and the internal verification group for the nomogram and AJCC TNM (8^th^) staging system of patients with pancreatic adenosquamous carcinoma. **(A)** 1-year development group **(B)** 2-year development group **(C)** 1-year internal verification group **(D)** 2-year internal verification group. **(E, F)** The 1-year and 2-year overall survival Decision Curve Analysis (DCA) of the nomogram and AJCC TNM (8^th^) staging system of patients with pancreatic adenosquamous carcinoma. **(E)** 1-year overall survival **(F)** 2-year overall survival. AJCC, American Joint Committee on Cancer.

To assess the predictive value of the established nomogram, we attempted to compare the predictive efficacy with the AJCC TNM (8^th^) stage. Its C index in the development and internal validation cohort was 0.609 (95% CI: 0.554-0.664), 0.581 (95% CI: 0.500-0.663), respectively, inferior to the nomogram. As **Figure 2** shows, through analysis of the AUC of a different cohort by ROC curves analysis, we also observed that the AUCs of the nomogram for 1-, 2- OS were better than those of the TNM stage in the development and validation cohort. (nomogram vs TNM, development cohort: 1 year, 0.750 vs 0.663; 2 year, 0.703 vs 0.626) (**Figures 2A–D**). The Decision Curve Analysis (DCA) also showed that compared with the AJCC TNM (8^th^) staging system, the predictive efficacy of the new nomogram is significantly increased and has a wide range of threshold probabilities both in the development and validation cohort (**Figures 2E, F**). These results indicated that the nomogram could be more beneficial in the clinical application of predicting individual survival outcomes than the AJCC TNM (8^th^) staging system.

### Performance of the Nomogram on external verification in a multi-center cohort from China

**Figure 3:**
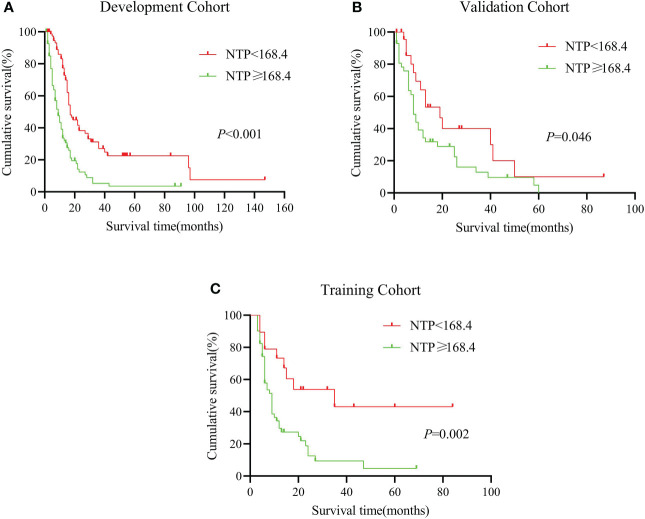
**(A–C)**: Kaplan-Miere analysis between different nomogram total scores predicting patients with pancreatic adenosquamous carcinoma in the **(A)**: development cohort, **(B)**: internal validation cohort, **(C)**: external validation cohort.

In order to judge the clinical applicability to other populations, we calculated the total nomogram point (NTP) and got the median number of 168.4 in the development group. Then we divided the cohorts into two subgroups according to the NTP, the low-risk group (NTP < 168.4) and the high-risk group (NTP ≧ 168.4). Moreover, there were 78 patients in the low-risk group and 87 patients in the high-risk group of the development cohort. As **Figure 3** shows, The Kaplan-Meire analysis showed that the low-risk group in the development cohort had a better prognosis than the high-risk group (*P* < 0.01). Similar results were also verified in the internal validation cohort.

Next, we put our multi-center cohort (n = 70) from Shanghai Ruijin Hospital and Xi’an Jiaotong University into the model. According to the NTP, we divided the cohort into low-risk and high-risk groups (n = 19) and high-risk groups (n = 51). According to the result, the median survival time in the two groups was 35 months (the low-risk group) vs. 9 months (the high-risk group). The 1-year survival is 67.2% vs. 29.7%, similar to the internal cohort analysis. It seemed that the established nomogram had an excellent performance in the external validation and could be widely suggested.

## Discussion

Pancreatic cancer is the fourth leading cause of cancer-related deaths in the United States, with 60,430 new cases and 68,220 deaths estimated in 2021 (12, 13). According to 2010 WHO classification, ASCP was classified as one subtype of PDAC (14). Previous studies consistently reported the survival or therapy of ASCP. One SEER analysis (4) compared the survival following surgical resection in patients with adenosquamous carcinoma or adenocarcinoma and the biological behavior and survival of ASCP and PDAC. Another analysis (7) focused on the benefit of chemoradiotherapy in ASCP treatment. In patients who underwent surgery, ASCP had worse OS than PDAC unless there was negative lymph node status, R0 surgical resection, and receipt of chemotherapy (15). There are relatively few models available for predicting survival outcomes of patients with ASCP.

As for PDAC, the clinician often makes empirical judgments according to the patients’ characteristics, AJCC stage, TNM stage, and pathological results. There are no specific methods for ASCP, in which all the TNM and AJCC staging criteria were based primarily on PDAC. Compared with the traditional predictive method, the nomogram could be more quick, convenient, and accurate (16). Their predictive value has been reported to be better than other evaluation systems and has been widely used in the study of various kinds of diseases (17–21). Here we developed a nomogram especially for ASCP to predict the survival and prognosis. We collected the relative index through univariate and multivariate analysis and incorporated these four parameters into the nomogram. By comparing the nomogram’s efficiency with the TNM stage, it was proved that the nomogram is superior to the TNM stage in predicting the prognosis of ASCP. Finally, we applied the external cohort from multi-center to verify the performance and get satisfactory results.

As to the poor prognosis of ASCP, Yuan Fang et al. (7) discovered that T staging, M staging, and adjuvant treatment, including chemo and radiotherapy, might be the indicator of survival benefits after ASCP resection. Ning Pu et al. (12) proposed a novel nomogram that included the T classification and LNR in patients with resected pancreatic head carcinoma. Additionally, some research showed that larger tumor sizes showed a shorter median OS than the T stage (15, 22). In recent years, LNR has been considered a robust predictor of survival in PC patients better than positive lymph nodes and an independent prognostic factor for patients after resection of pancreatic cancer (23–25). You et al. (26) reported that LNR showed the best prognostic performance and a significant relationship with locoregional recurrence in pancreatic cancer treated with R0 resection and adjuvant treatment. Moreover, Patients affected by ASCP or SCC undergoing surgery and post-operative treatment with chemotherapy, radiotherapy, or both appear to benefit, even though there is no consensus regarding the best regimen to use, which are commonly used fluoropyrimidine-based, gemcitabine-based, or platinum-based. Data from Johns Hopkins Hospital show, in particular, benefit for ASCP patients treated with platinum-based chemotherapy, with an OS of 19.1 months (27).

Using this nomogram, we may predict the future survival rate of the patients more accurately. However, the C-indexes and AUCs of the development and validation cohort nomogram were more accurate than the current TNM staging in predicting the prognosis. Further, DCA demonstrated its clear clinical application advantages over the TNM staging system. To further prove the efficiency of the nomogram, we applied two Chinese centers to verify the nomogram. It is convincing that the results of this study could be particularly helpful in predicting post-operative survival of ASCP patients.

Overall, the nomogram is innovative and reasonable in the following aspects. Firstly, variables like tumor size, chemotherapy, radiotherapy, and LNR were used to develop this nomogram. Secondly, the nomogram based on the SEER database was able to predict the prognosis of ASCP. ROC curve and DCA analyses of this study showed that the nomogram could predict the OS of patients more accurately, which has clinical applicability. The external validation of nomogram prediction from the multi-center cohort was found to be accurate.

The limitation was also considered in our study. First, though this retrospective study uses the SEER database and two medical institutions, the sample of the external cohort is small, and a multi-center prospective study is needed to increase the number of cases further to improve the accuracy and representativeness of the prediction model. We developed a nomogram of adenosquamous carcinoma of the pancreas (ASCP) based on retrospective studies of the SEER database, which required further validation in prospective cohort and clinical trials. In addition, this study did not include information on the gene targets and molecular markers. Targeting the tumor microenvironment may play an essential role in the therapeutic strategies of PDAC and rare pancreatic tumors (28, 29). Molecular biology, genetics, and epigenetics provide new evaluation indicators of individual rare pancreatic neoplasms’ potential behavior. Compared with PDAC, more and more relevant studies of ASCP focused on the analysis of molecular features and genetic alterations of ASCP (30, 31). For example, Lenkiewicz E et al. (30) found that ASCP organoids were carrying an FGFR1 fusion show sensitivity to pan FGFR inhibitor (infigratinib), the first example of ASCP response to targeted therapy. Above all, new biomarkers and genetic alterations could be added to future prediction models of ASCP to provide more accurate individual risk estimations. Since there is limited literature related to ASCP, if possible, we expect to distinguish the genomic and epigenomic landscape of ASCP and identify new strategies for targeting this aggressive subtype of pancreatic cancer. Molecular profiling of ASCP may be appropriate to provide complete information regarding the patient’s tumor. Tumor microenvironment and molecular features of ASCP could be our next research topic. Considering this, the nomogram prediction model established in this study can be used for future research.

## Conclusion

Here we developed a nomogram especially for ASCP to predict the survival and prognosis. Univariate and multivariate analysis showed that four parameters, including tumor size, radiotherapy, chemotherapy, and LNR, could influence the survival of ASCP patients. In addition, by comparing the nomogram’s efficiency with AJCC TNM (8^th^) staging system, it was proved that the nomogram is superior to the TNM stage in predicting the prognosis. Finally, we applied the external cohort from multi-center to verify the performance and get satisfactory results. Our nomogram has clinical applicability.

## Data availability statement

The raw data supporting the conclusions of this article will be made available by the authors, without undue reservation.

## Ethics statement

The studies involving human participants were reviewed and approved by The Institutional Review Boards of the First Affiliated Hospital of Xi’an Jiaotong University. Written informed consent for participation was not required for this study in accordance with the national legislation and the institutional requirements.

## Author contributions

Conception and design: ZWa, YS, ZWu, and QM. Administrative support: ZWang, ZWu, QM, XY, and LH. Provision of study materials or patients: CR, ZWang, JJ, and YS. Collection and assembly of data: CR, YM, JJ, JD, YJ, YW, and ZWang. Data analysis and interpretation: CR, YM, ZWang, WL, YS, JD, YJ, and YW. Manuscript writing: all authors. Final approval of manuscript: all authors.

## Funding

This study was financially supported by the National Natural Science Foundation of China (NSFC 81872008 to ZW), the Science and Technology Innovation as a Whole Plan Projects of Shaanxi Province, China (No. 2016 KJZDSF01-05-01), and Shaanxi Provincial Key Research and Development Project. (No. 2018 KW-064).

## Acknowledgments

The authors thank all colleagues who contributed to this effort.

## Conflict of interest

The authors declare that the research was conducted in the absence of any commercial or financial relationships that could be construed as a potential conflict of interest.

## Publisher’s note

All claims expressed in this article are solely those of the authors and do not necessarily represent those of their affiliated organizations, or those of the publisher, the editors and the reviewers. Any product that may be evaluated in this article, or claim that may be made by its manufacturer, is not guaranteed or endorsed by the publisher.
