# Supplementary material for: Development and external validation of a dynamic nomogram to predict the survival for adenosquamous carcinoma of the pancreas
Source: Front Oncol. 2022 Aug 12;12:927107. doi: 10.3389/fonc.2022.927107 (PMC9411813; doi:10.3389/fonc.2022.927107)
Supplement: Supplementary file 1 [file DataSheet_1.docx]

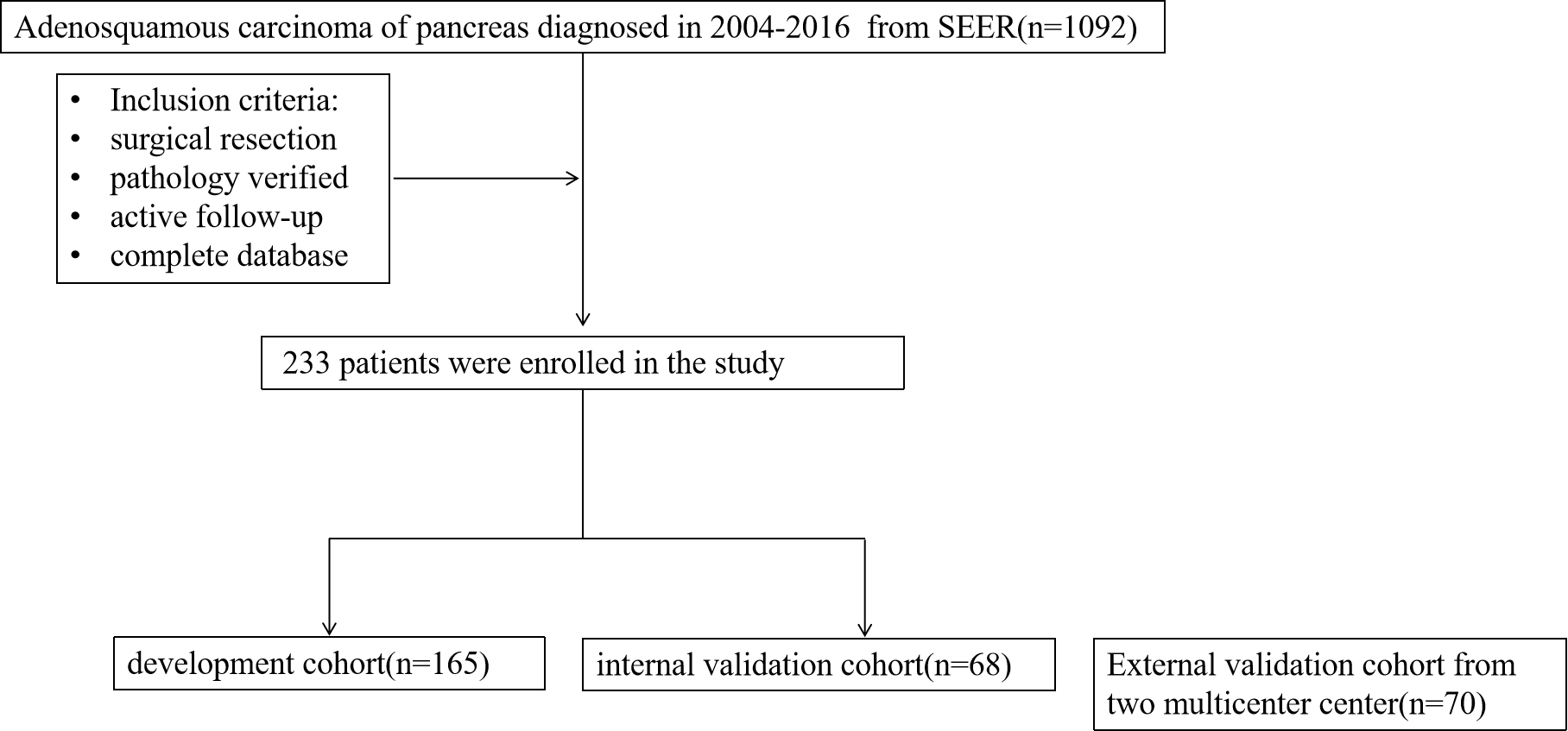


Figure S1: Screening process of patients with pancreatic adenosquamous carcinoma in the study


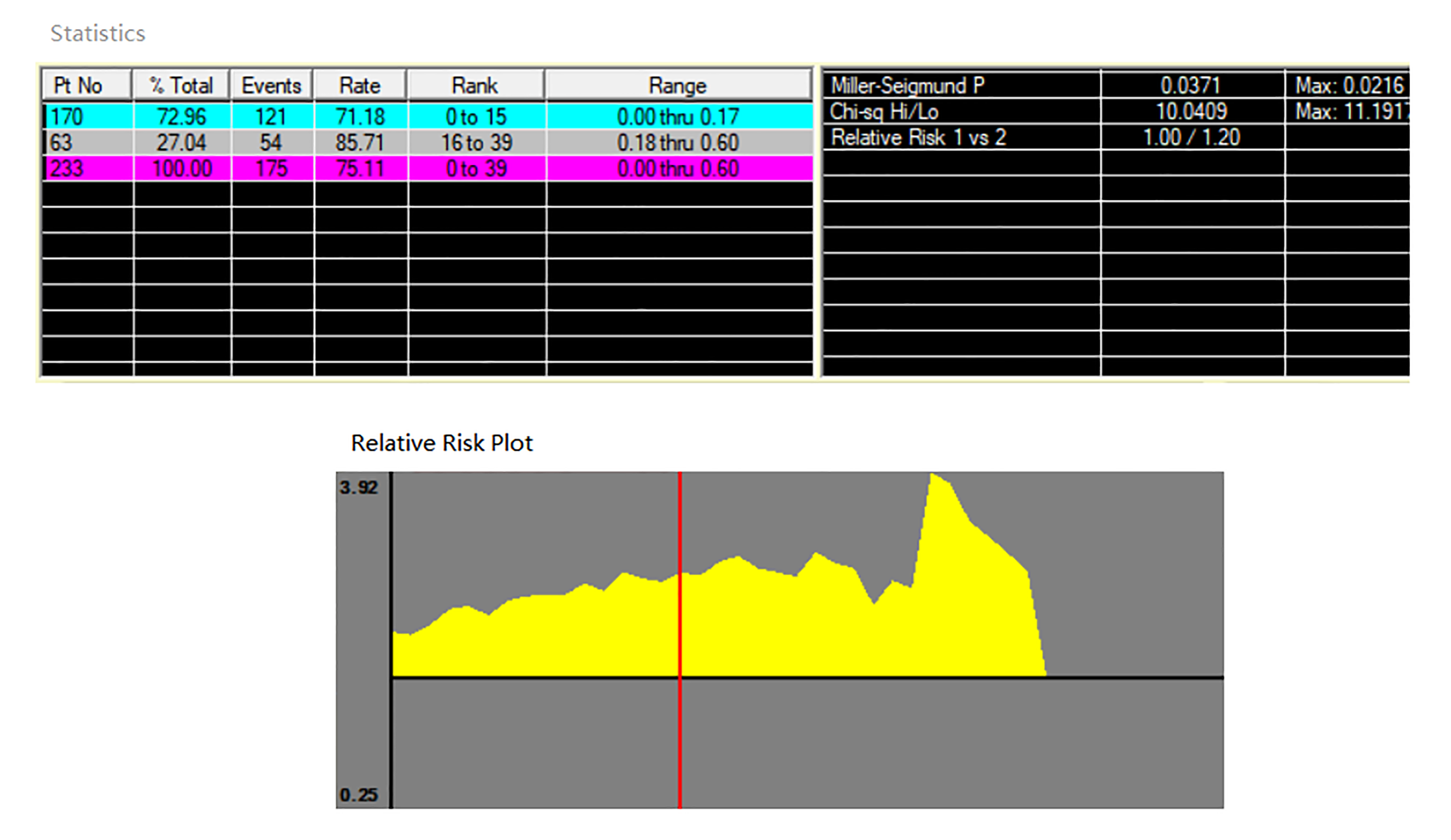


Figure S2: X-tile analysis for The optimal cutoff value of lymph nodes ratio of patients with pancreatic adenosquamous carcinoma in the study


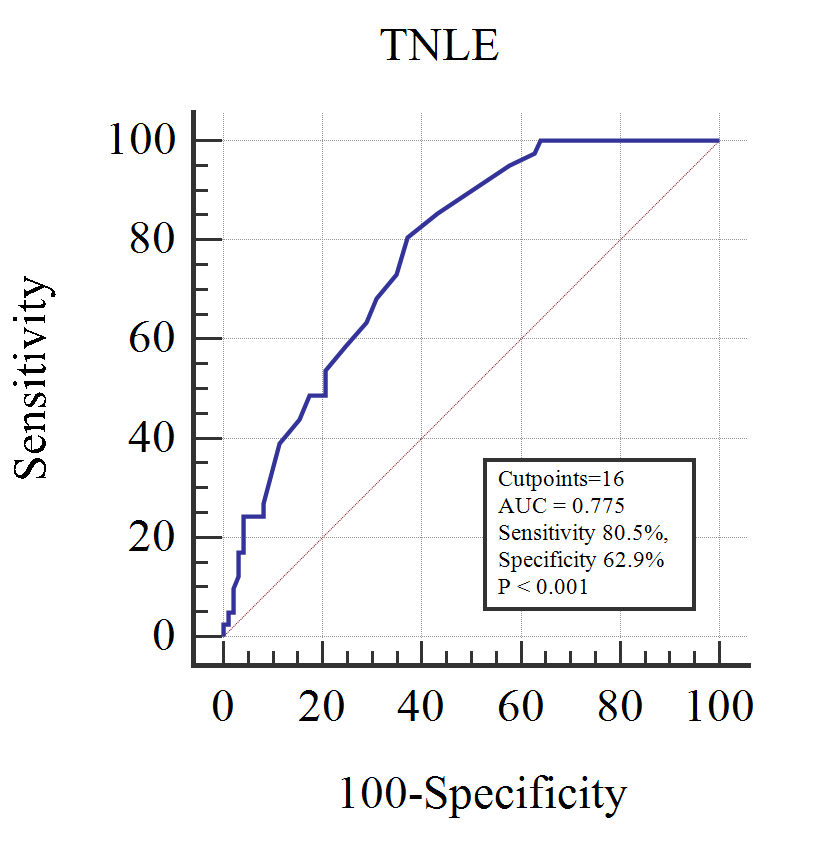


Figure S3: Receiver-operating characteristic (ROC) analysis illustrating that total number of lymph nodes examined (TNLE) ≧16 had the highest discriminatory power relative to overall survival among patients who had 1 to 3 lymph node metastasis (LNM) and patients who had≧4 LNM (AUC 0.775, Youden index 0.434, sensitivity 80.5%, specificity 62.9%, *P* < 0.001).


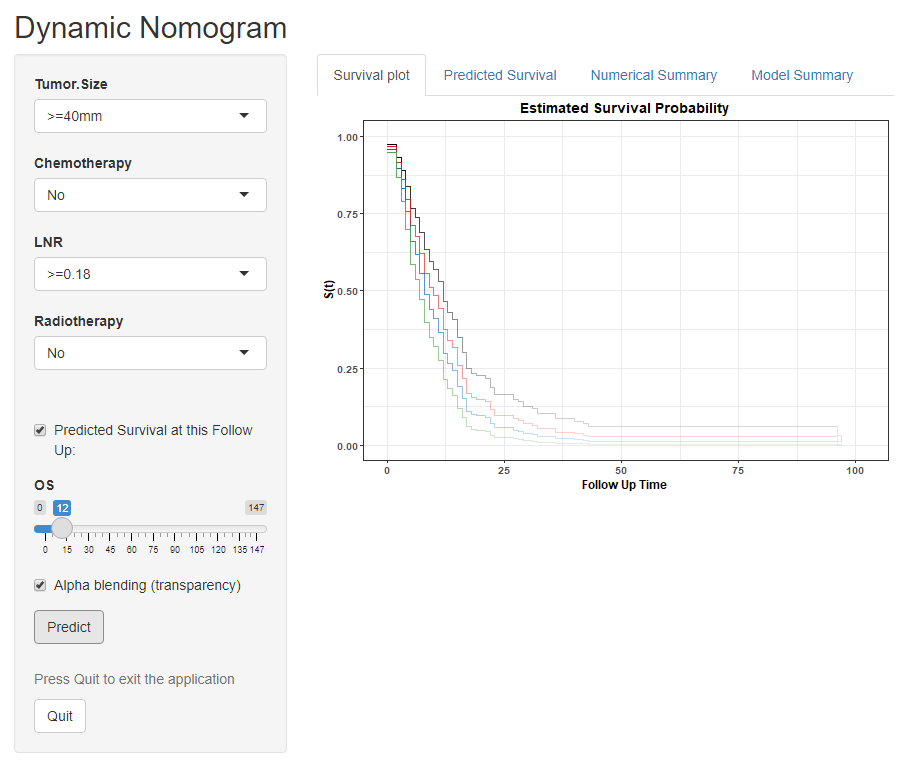


Figure S4: A sample demonstration of the Dynamic nomogram of patients with pancreatic adenosquamous carcinoma in the study
